# Stimulus-Responsive Modulation of Solvation Environments in Solid Catalysts

**DOI:** 10.1021/acs.accounts.5c00576

**Published:** 2025-11-10

**Authors:** Pengcheng Huang, Bin Wang, Jimmy A. Faria Albanese

**Affiliations:** † Jiangsu Key Laboratory of Advanced Catalytic Materials and Technology, School of Petrochemical Engineering, Changzhou University, Changzhou 213164, P. R. China; ‡ School of Sustainable Chemical, Biological and Materials Engineering (SCBME), 6187 University of Oklahoma, Norman, Oklahoma 73019, United States; § Catalytic Processes and Materials Group, Faculty of Science and Technology, MESA+ Institute for Nanotechnology, 3230 University of Twente, P.O. Box 217, 7500 AE Enschede, The Netherlands

## Abstract

Liquid environments play a crucial role in the biological processes occurring in living organisms as well as in many human-made processes involving electrochemistry, photo-, and thermocatalysis. In the majority of these systems, aqueous phases are ubiquitous due to water’s natural abundance. Water molecules, however, can exert large changes in the chemical environment of catalytically active sites, altering the reaction rates, selectivity, and catalyst stability. These solvation effects induced by water molecules near catalytic sites can drastically change the energy landscape and unlock unique reaction pathways with far more favorable kinetics. In nature, living organisms couple these complex interactions with detection, communication, and actuation mechanisms to induce self-regulatory behavior, ensuring stability of the system and thus long-term durability. Extrapolating this behavior to heterogeneous catalysis is desirable because the resulting “smart materials” can potentially unlock new chemical conversion processes with higher atom efficiency, rates, and stability.

The combination of polymer chemistry and heterogeneous catalysis has introduced versatile approaches for creating materials that can respond to cues in the reaction medium that alter the accessibility, intrinsic activity, and selectivity of the catalyst. To achieve this, one could combine stimulus-responsive polymers, which undergo a large volumetric phase transition in response to an external stimulus, with a solid catalyst. This chemo-mechanical response has been employed to create a variety of nanoreactor vessels with stimulus-responsive character that turn on- and off- depending on the reaction conditions. In this Account, we focus on the impact of these polymer coatings on the solvation environment around the active site and the implications of these effects on the reaction energy landscape, molecular arrangement of the solvent, electric fields at the catalyst–liquid interface, binding energy, and mobility of surface reaction intermediates. These seemingly subtle changes in solvent molecules induced by the presence of polymers can have a tremendous impact on the development of bioinspired heterogeneous catalysts, reliable chemical clocks, micro/nanoreactors, and robots. The large library of polymer chemistries offers a plethora of combinations of stimulus-responsive mechanisms (e.g., temperature, pH, light, magnetic field, solvent composition), providing the possibility of creating homeostatic catalysts *à la carte*.

## Graphical abstract

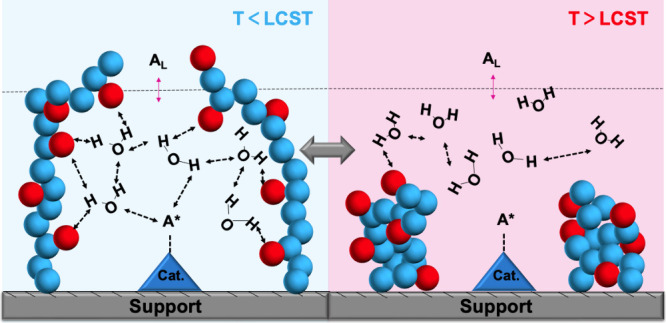

## Key References

-

Huang, P. ; Baldenhofer, R. ; Martinho, R. P. ; Lefferts, L. ; Faria Albanese, J. A. Stimulus-Responsive Control of Transition States on Nanohybrid Polymer–Metal Catalysts. ACS Catal. 2023, 13, 6590–6602 .^1^ *This work provides for the first time a mechanistic description of solvation effects induced in an ON/OFF fashion by stimulus-responsive polymers during the reduction of nitrobenzene on Pd/SiO_2_ catalysts.*
-

Huang, P. ; Yan, Y. ; Martinho, R. P. ; Lefferts, L. ; Wang, B. ; Faria Albanese, J. Water Confinement on Polymer Coatings Dictates Proton–Electron Transfer on Metal-Catalyzed Hydrogenation of Nitrite. JACS Au 2024, 4, 2656–2665 39055155 10.1021/jacsau.4c00389PMC11267551.^2^ *This work demonstrated that water confined in polymeric films can induce reversible changes in the microsolvation environment near the surface of metal catalysts. The confined water strongly interacts with p-NIPAM polymers, which decreases the strong electric fields induced by the presence of water molecules near the metal surface.*
-

Huang, P. ; Betting, J. ; Tian, S. ; Lefferts, L. ; Faria Albanese, J. Modifying Reaction Rates and Stimulus-Responsive Behavior of Polymer-Coated Catalysts Using Aprotic Solvents. J. Catal. 2023, 428, 115157 .^3^ *These results indicate that p-NIPAM polymers covalently bonded to the catalyst surface can exert drastic changes in the energy landscape of catalytic reactions when the solvent medium contains water molecules that interact via hydrogen bonding with both chemisorbed hydrogen and p-NIPAM polar moieties.*
-

da Silva, M. J. E. ; Lefferts, L. ; Faria Albanese, J. A. 
*N*-Isopropylacrylamide Polymer Brushes Alter the Micro-Solvation Environment during Aqueous Nitrite Hydrogenation on Pd/Al_2_O_3_ Catalyst. J. Catal. 2021, 402, 114–124 .^4^ *This contribution shows that polymer brushes modify the selectivity of supported palladium catalysts during the nitrite hydrogenation reaction. p-NIPAM brushes effectively induce solvation effects that modify the energy landscape of the catalytic reaction, mimicking the operation of enzymes.*

## Introduction

Creating artificial materials that can mimic the responsiveness, activity, and selectivity of enzymes has captivated the scientific curiosity of researchers working on homogeneous and heterogeneous catalysis. In living organisms, the enzymatic pocket containing the active site has evolved to direct specific chemical transformations, leveraging the molecular interactions between the reactant, the solvent environment, and the chemical moieties of the enzyme.^5–7^ This delicate balance of intra- and intermolecular interactions creates kinetically favorable reaction pathways that allow activation of highly stable reactants (e.g., N_2_, CO_2_, H_2_O) at mild temperatures.

In heterogeneous catalysts, achieving the activity, precision, and responsiveness observed in enzymes is rather challenging because of the rigid nature of the materials that compose the catalyst. To bridge this gap, one could introduce a flexible functionality that provides stimulus-responsiveness to the catalyst.^8–12^ This can be used to modify in a reversible manner the mass transfer rates to the active site, intrinsic activity of the catalytic center, or the solvation environment. The archetypal example of stimulus-responsive catalysts is the temperature-responsive poly­(*N*-isopropylacrylamide) (p-NIPAM)-functionalized hydrogels containing metal active sites.^13^ Prof. M. Ballauff and Prof. J. Dzubiella pioneered the use of Debye–Smoluchowski theory to link p-NIPAM responsiveness with catalytic activity with negligible deactivation or change in selectivity. They showed that observed kinetics (*k*_obs_) results from coupling diffusion (*k*_d_) and intrinsic reaction (*k*_r_),^14^ expressed as

1 $${k_{\mathrm{obs}}}^{-1}={k_{r}}^{-1}+{k_{d}}^{-1}$$

At low temperature, swollen hydrogels enhance the permeability and solubility of polar reactants, so *k*_obs_ approaches *k*_r_.^15^ When the polymer collapses, diffusion dominates, so *k*_obs_ resembles *k*_d_.^16–18^ Notably, the opposite will occur for hydrophobic species. In that case, the collapse promotes nonpolar interactions and thus higher rates. In contrast, below the lower critical solution temperature (LCST) the polymer is hydrophilic, reducing partitioning of nonpolar species and thus the flux, despite high porosity.^18^ The LCST is a key property in polymer solutions, as it describes the temperature at which a soluble polymer precipitates out of solution.

This description has several caveats. When stimulus-responsive polymers are covalently anchored to the surface of a porous catalyst, the interplay between pore size and polymer brush length governs the system’s behavior. If the brushes are shorter than the pores, the solvated polymer can fill the pores, increasing the diffusion resistance and lowering the reaction rates. Upon heating, the brushes collapse, reducing the polymer-occupied pore volume and accelerating diffusion. In contrast, if the brushes are longer than the pores, the polymer layer and the porous support act as two resistances in series, where the polymer anchored on the external surface of the catalyst serves as a gate regulating diffusion. In this scenario, at low temperatures, when the polymer is solvated, diffusion is unrestricted, and the kinetics are fast, while the opposite occurs at high temperatures. This key effect is absent from the proposed model. Second, if the catalyst support particles are sufficiently small, then the system operates under kinetic control. In this regime, changes in polymer solvation state do not affect performance, as mass transport resistance is negligible compared to intrinsic kinetics. As a result, the apparent stimulus-responsiveness disappears. Only when support particles are large enough for mass transfer to approach a limiting regime does stimulus-responsive behavior become evident.^19^ Finally, eq 1 assumes that the intrinsic kinetic constant of the bare catalyst is identical to that of the polymer-coated catalyst in its solvated state. However, experiments conducted under conditions free of mass transport limitations demonstrate that these polymers can significantly alter apparent activation barriers, pre-exponential factors, product selectivity, and degrees of rate control. Since neither external nor internal mass transport can account for these variations, they must arise from solvation effects.^1–4^

In this Account, we review the underlying chemistry and physics that control the solvent–polymer interactions and the interplay between the catalyst performance and the solvation layer. We will discuss the main criteria that must be considered when designing and studying these materials to ensure that solvation effects, rather than mass transfer limitations, control the observed responsive behavior. Then, we explore different examples that showcase the application of these stimulus-responsive catalysts to modify the solvation environment near catalytic sites. Finally, an outlook on the concept will be discussed as well as its implications in the fields of biomass conversion, removal of contaminants in water, electrochemistry, and photochemistry.

## Designing Rules for Stimulus-Responsive Modulation of Solvation Environments

To design a responsive catalyst in which the changes in the reaction rate are induced by reversible modifications of the solvation environment, one must ensure that the following precepts are followed:

(i) the reaction is free of mass transport limitations (porous vs nonporous catalysts) in the two states of the polymer;
(ii) the polymer is stable (i.e., no lixiviation or thermal degradation);
(iii) the polymer solvation state must be fully reversible and reproducible;
(iv) the volumetric phase transition must be sharp and large to alter the interactions between the solvent and the reaction intermediates;
(v) the polymer coating density and polymer length must be sufficiently large to modify the interactions between the solvent molecules and the key reaction intermediates.

## Translating Mechanochemical Response into Solvation-Catalytic Actuation

Adding polymers or other functionalities to a catalyst surface reorganizes the local solvation environment, which can alter activation barriers, reaction orders, selectivity, and activity. When the polymer shifts from a solvated to a collapsed state, the extent of solvation changes, and with it, catalyst performance. Transition-state treatments allow these polymer-induced effects to be separated from those of the unmodified liquid phase. In this framework, the rate per site (*r*/[L]) depends on the transition-state crossing frequency (ν) at a given temperature and the concentration of the transition-state complex for the reaction A → B (eq 2):

2 $$\frac{r}{\left[L\right]}=\nu {\left[A\right]}^{⧧}$$

The vibrational frequency can be obtained from density functional theory (DFT) or approximated as *k*_B_ *Th* ^–1^, while the concentration of the transition-state complex arises from the equilibrium between reactants and the transition state (eq 3):

3 $$A^{*}⇄{\left[A\right]}^{⧧}\to B$$

This equilibrium depends on the chemical potentials of the reactants in the liquid, described by their activity coefficients and concentrations. For gas–liquid systems, concentrations can be derived from Henry’s law; for charged species, electrostatic interactions and polarizability near the active site must also be considered. Neglecting electric fields, the rate expression includes the ratio of the activity coefficients of reactants and the transition state (eq 4):

4 $$\frac{r}{\left[L\right]}=\frac{k_{B}T}{h}K^{⧧}K_{A}\frac{\gamma_{A}}{\gamma^{⧧}}\frac{\left[A\right]}{1+{K_{A}\gamma }_{A}\left[A\right]}$$

where *K*_A_ is the adsorption constant of reactant A, *K* ^⧧^ is the equilibrium constant for the transition state, and γ_A_ and γ^⧧^ are the activity coefficients of A and the transition state, respectively. Since $\frac{\gamma_{A}}{\gamma^{⧧}}$ will be equal to unity only in rare cases, solvation effects are essentially unavoidable in liquid systems. Two distinct modes of interaction can be identified between stimulus-responsive polymers and catalysts. In the direct transduction mode, the polymer’s response to external stimuli directly modifies the solvation environment of the catalytic sites, influencing activation barriers through solvent–transition state interactions. In the indirect transduction mode, the polymer’s response alters the binding energies of the species prior to the transition state as well as the activated complex.

### Direct Transduction of Stimulus Response to Catalytic Solvation Effects

In this case, the surface is saturated by the reactant, which results in a rate with a zeroth-order dependence on the concentration of the reactant (eq 5):

5 $$\frac{r}{\left[L\right]}=\frac{k_{B}T}{h}\frac{K^{⧧}}{\gamma^{⧧}}$$

In direct transduction, solvents may affect the activation barriers through interaction with the transition states, manifested by changes in the equilibrium constant and activity coefficient of the transition state. Accurate estimation of the activity coefficients of the reactants in the liquid phase can be done by using a variety of experimental and theoretical methods. Determining this quantity for the transition state, however, is not trivial, as it requires a molecular description of the kinetically controlling reaction step and the liquid–solid interface, which is done using computationally demanding ab initio molecular dynamics (AIMD) simulations and enhanced sampling approaches. To circumvent this limitation, one could use the kinetics at saturated conditions to directly determine the extent of solvation, allowing the direct transduction of the stimulus response of the polymer into a catalytic solvation effect. For this, one can rewrite the rate expression shown in eq 5 in terms of the excess Gibbs free energy (γ_*i*_ = exp­[(*G*_*i*_^ε^)/*RT*]), giving the equation shown below (eq 6):

6 $$\frac{r}{\left[L\right]}=\frac{k_{B}T}{h}\frac{e^{-{\Delta G}^{{}^{\circ},⧧}/RT}}{e^{G^{\varepsilon ,⧧}/RT}}$$

This can be rearranged to generate a rate expression in which all of the solvation effects associated with the changes in the presence of the polymer are captured by an apparent excess Gibbs free energy change (eq 7):

$$\frac{r}{\left[L\right]}=\frac{k_{B}T}{h}e^{\left(-\frac{{\Delta G}^{{}^{\circ},⧧}+G^{\varepsilon ,⧧}}{RT}\right)}$$ 7

This means that the nonidealities of the reactant A in the liquid phase are relevant for the observed changes in the reaction rate. Under these conditions, one can directly isolate the impact of the stimulus-responsive polymer on the reaction rate by measuring the rates at different temperatures and saturation kinetics. Using the van’t Hoff plot of the equilibrium constant for the transition state (*K* ^⧧^), one would be able to determine the enthalpic and entropic contributions to the Gibbs free energy of the transition state at two limiting conditions: one for the high-temperature region when the polymer is solvated and one for the collapsed state at high temperatures. Under these conditions, the changes in the response of the reaction rate with the temperature will be associated with the enthalpic excess energy induced by the polymer at the solvated (i) and collapsed (ii) states, while the alterations in the intrinsic activity at a given temperature will be given by the entropic contributions (eqs 8 and 9):^7,20,21^

$$\frac{r_{i}}{\left[L\right]}=\frac{k_{B}T}{h}e^{\left(\frac{{\Delta S}^{{}^{\circ},⧧}+S^{\varepsilon ,⧧,i}}{R}\right)}e^{\left(-\frac{{\Delta H}^{{}^{\circ},⧧}+H^{\varepsilon ,⧧,i}}{RT}\right)}$$ 8

$$\frac{r_{\mathrm{ii}}}{\left[L\right]}=\frac{k_{B}T}{h}e^{\left(\frac{{\Delta S}^{{}^{\circ},⧧}+S^{\varepsilon ,⧧,\mathrm{ii}}}{R}\right)}e^{\left(-\frac{{\Delta H}^{{}^{\circ},⧧}+H^{\varepsilon ,⧧,\mathrm{ii}}}{RT}\right)}$$ 9

These effects will directly alter the solvation of the transition state.

### Indirect Transduction of Stimulus Response to Catalytic Solvation Effects

Here, the catalyst surface is empty, which leads to a first-order kinetic expression in which the adsorption constant of the reactant and its solvation in the liquid phase are part of the rate. Now, the rate can be written as a function of the apparent reaction equilibrium constant for the transition state *K*_app_^⧧^= *K* ^⧧^ *K*_A_ (eq 10):

10 $$\frac{r}{\left[L\right]}=\frac{k_{B}T}{h}K_{\mathrm{app}}^{⧧}\frac{\gamma_{A}}{\gamma^{⧧}}\left[A\right]$$

In contrast to the previous case, the apparent barrier is coupled to the changes in the adsorption energetics of the reactant A as well as the solvation of the transition state itself.

Applying the same definition of the activity coefficients that we employed in eq 6, one can obtain an expression in which the excess Gibbs free energy of activation becomes part of the rate expression, as the ratio γ_A_/γ^⧧^ is equal to exp­(−Δ*G*_app_^ε,⧧^/*RT*). This results in a rate equation that contains the change in the excess Gibbs free energy of apparent activation (eq 11):

$$\frac{r}{\left[L\right]}=\frac{k_{B}T}{h}e^{\left(-\frac{{\Delta G}_{\mathrm{app}}^{{}^{\circ},⧧}+{\Delta G}_{\mathrm{app}}^{\varepsilon ,⧧}}{RT}\right)}\left[A\right]$$ 11

If one would determine the reaction kinetics at different temperatures at low coverages of the reactants, then it would be possible to transduce the stimulus response of the polymer into a change in the solvation environment of the catalyst in an indirect manner, as the changes observed in the apparent enthalpy and entropy of activation would be associated with both the state of the solvation of the reactants and that of the transition state (eq 12):

$$\frac{r}{\left[L\right]}=\frac{k_{B}T}{h}e^{\left(\frac{{\Delta S}_{\mathrm{app}}^{{}^{\circ},⧧}+{\Delta S}_{\mathrm{app}}^{\varepsilon ,⧧}}{R}\right)}e^{\left(-\frac{{\Delta H}_{\mathrm{app}}^{{}^{\circ},⧧}+{\Delta H}_{\mathrm{app}}^{\varepsilon ,⧧}}{RT}\right)}\left[A\right]$$ 12

Here, two states of solvation of the polymer would lead to distinct states of interaction between the solvent and reactants, which in turn will result in different reaction kinetics in the solvated (i) and collapsed (ii) states of the polymer (eqs 13 and 14):

$$\frac{r_{i}}{\left[L\right]}=\frac{k_{B}T}{h}e^{\left(\frac{{\Delta S}_{\mathrm{app}}^{{}^{\circ},⧧}+{\Delta S}_{\mathrm{app}}^{\varepsilon ,⧧,i}}{R}\right)}e^{\left(-\frac{{\Delta H}_{\mathrm{app}}^{{}^{\circ},⧧}+{\Delta H}_{\mathrm{app}}^{\varepsilon ,⧧,i}}{RT}\right)}\left[A\right]$$ 13

$$\frac{r_{\mathrm{ii}}}{\left[L\right]}=\frac{k_{B}T}{h}e^{\left(\frac{{\Delta S}_{\mathrm{app}}^{{}^{\circ},⧧}+{\Delta S}_{\mathrm{app}}^{\varepsilon ,⧧,\mathrm{ii}}}{R}\right)}e^{\left(-\frac{{\Delta H}_{\mathrm{app}}^{{}^{\circ},⧧}+{\Delta H}_{\mathrm{app}}^{\varepsilon ,⧧,\mathrm{ii}}}{RT}\right)}\left[A\right]$$ 14

If the stimulus-responsive polymer has the same extent of interaction with the solvent nearby the active site in its two solvation states, then the resulting rates would be the same on both conditions.

We have shown that when N–O hydrogenation reactions, such as those involved in NO_2_^–^ and nitrobenzene reduction, are conducted in the aqueous phase, these polymer–reactant interactions are widely different at the two states of the polymer solvation (Figure 1). At the solvated state, the polymer interacts with the water molecules in the vicinity of the active site, leading to lower barriers, as there is a stabilization of the transition state. This generalized concept would suggest that when the polymer is solvated, the interactions with the water molecules near the active site facilitate the formation of the transition state in either one of the two methodologies (direct or indirect transduction). However, one must be aware that the opposite could happen if the interactions with either the kinetically relevant most abundant surface reaction intermediate or with the solvent molecules lead to destabilization interactions.

**Figure 1:**
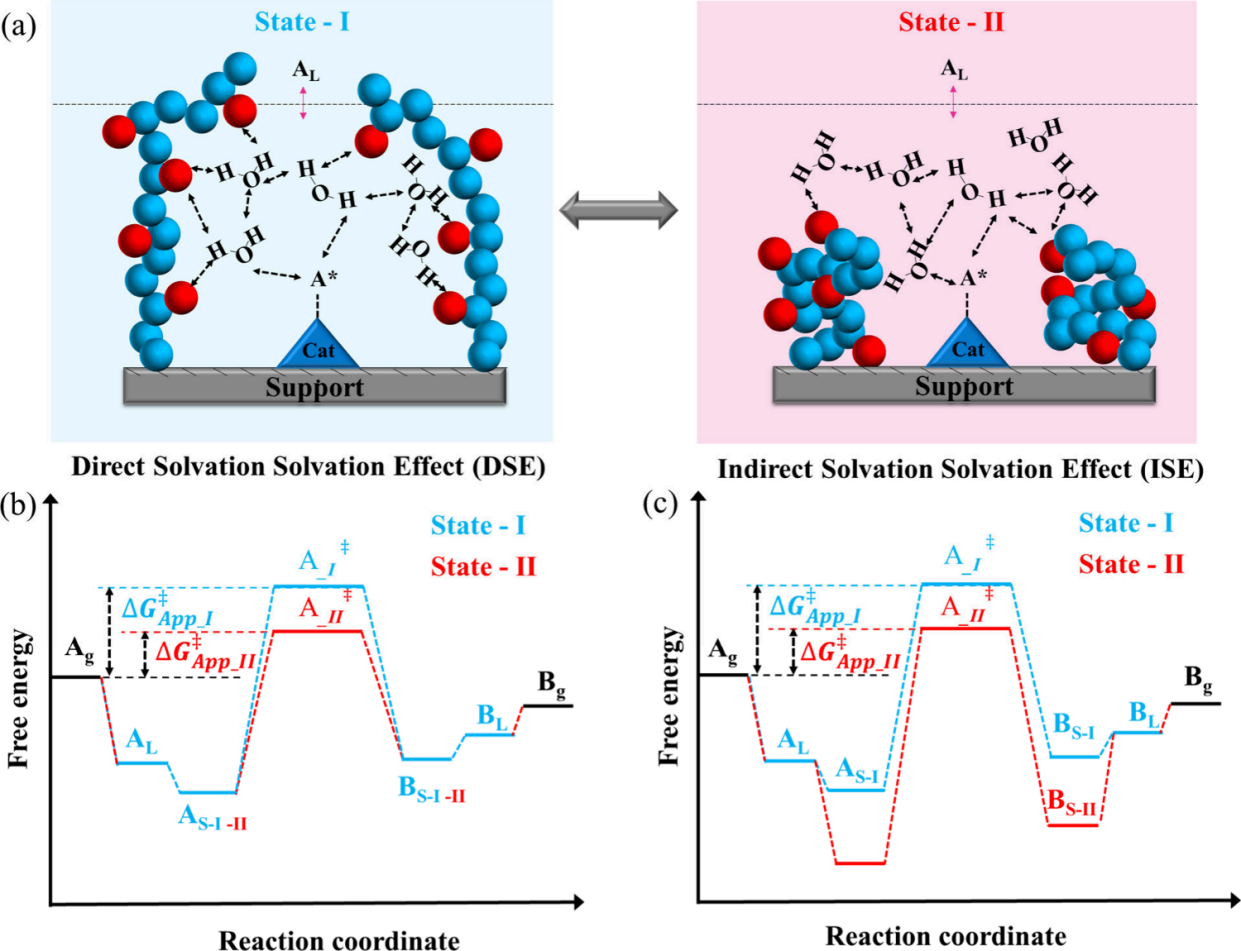
(a) Schematic illustration of the changes in the interaction between the water molecules of the solvent, the stimulus-responsive polymer, and the chemisorbed reactants (A*) in the solvated and collapsed states of the polymer, together with the two possible scenarios in which there is an enhancement in the reaction barrier as a result of the presence of the polymer influencing the energy landscape either (b) directly by changing the energetics of the transition state or (c) indirectly by altering the binding energies of the chemisorbed reactants.

## Synthesis of Stimulus-Responsive Polymer on Heterogeneous Catalysts

The most common strategies to create smart nanoreactors rely on the idea of creating a reversible mass transport limitation rather than changing the solvation. Therefore, careful tuning is required to generate a polymer coating that can allow unrestricted access for reactants and products to the active sites in the extended and collapsed states of the polymer while inducing a change in the reaction kinetics. In the next section, we describe our approach to generating these materials.

The process starts with the synthesis of spherical silica particles. By using the Stöber method for growing colloidal silica (SiO_2_) followed by metal deposition using strong electrostatic adsorption (SEA), it is possible to achieve high metal dispersion (Figure 2a). Since there are no pores on these particles, it is possible to avoid internal mass transport limitations. Then the material is functionalized using (3-aminopropyl)­triethoxysilane (APTES). This molecule readily reacts with the −OH moieties on silica to generate (Si–O)_3_Si­(CH_2_)_3_NH_2_ species that are stable up to 275 °C. Then stimulus-responsive brushes are grown from the surface via atom transfer radical polymerization (ATRP) using *N*-isopropylacrylamide as the monomer. The thickness of the polymer coating after 24 h of growth can reach values of up to 6 nm (Figure 2d). The particle size of the palladium clusters before and after the ATRP polymerization was essentially unaltered, with metal clusters with varying sizes between 1 and 2.5 nm.

**Figure 2:**
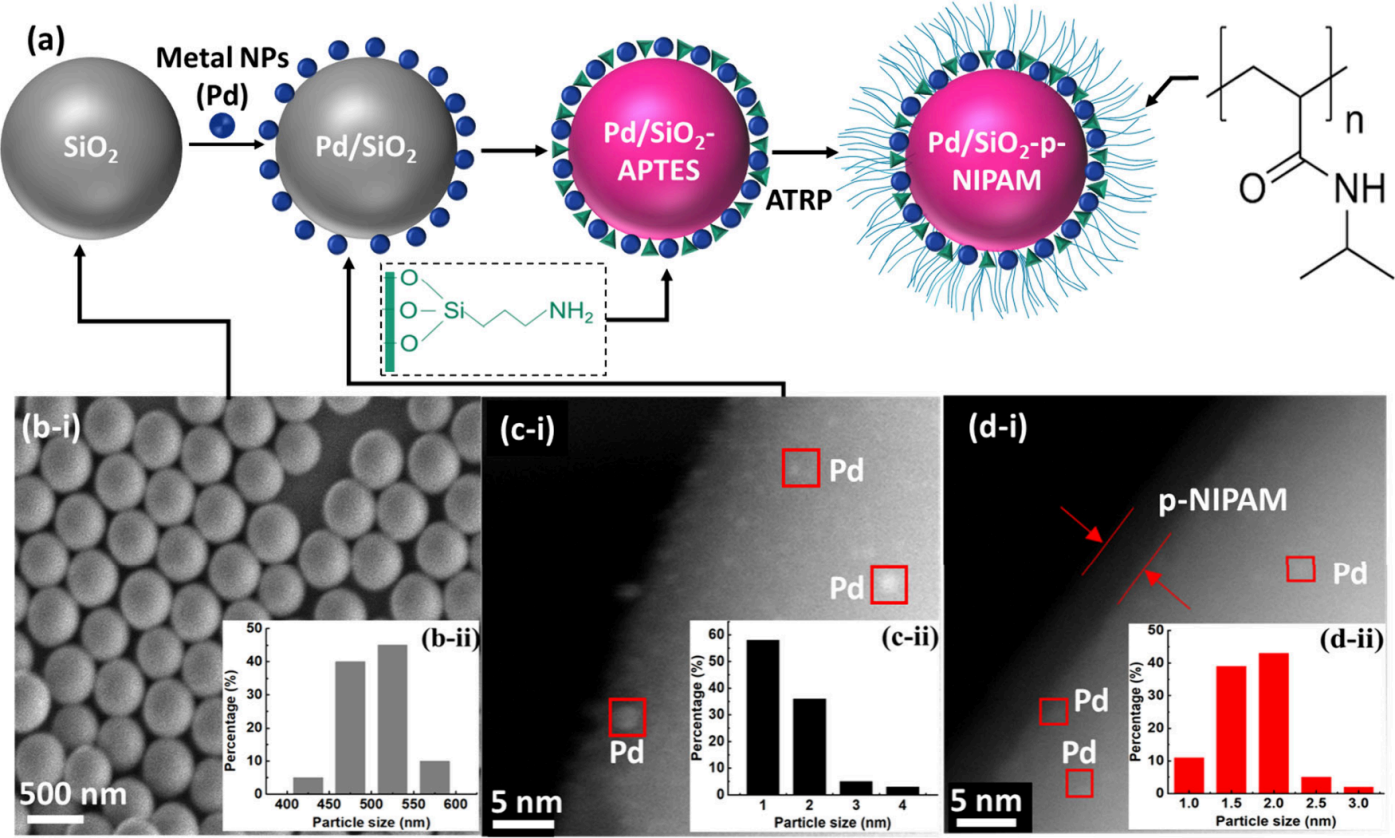
(a) Synthesis methodology based on growth of silica spheres using the Stöber process followed by metal deposition via strong electrostatic adsorption (SEA) and surface silylation to generate the anchoring for poly­(*N*-isopropylacrylamide) (p-NIPAM) polymeric brushes leveraging atom transfer radical polymerization (ATRP). (b-i) Scanning electron microscopy (SEM) of the Stöber SiO_2_ and (b-ii) particle size distribution of the SiO_2_. (c-i, d-i) Transmission electron microscopy (TEM) of the metal catalysts supported on the silica before and after polymer growth and (c-ii and d-ii) particle size distributions of Pd particles. Reproduced from ref 2. CC BY-NC-ND 4.0.

The properties of surface-anchored polymer brushes can be tuned by polymer type, anchoring points, grafting density, topology, and functionality.^22^ ATRP is particularly effective for its synthesis, offering precise control through reversible activation–deactivation of chain growth.^23,24^ The brush behavior depends on the grafting density (σ) and thickness (*H*):^25,26^ at low σ, chains adopt a mushroom regime, while higher σ induces brush stretching due to steric hindrance and osmotic pressure (Figure 3a).^26^ The swollen brush layer thus exceeds the radius of gyration (*R*_g_) of free chains.^27^ Stimulus-responsiveness is demonstrated by dynamic light scattering: the particle size decreases from ∼645 nm (swollen, <32 °C) to ∼510 nm (collapsed, >32 °C), indicating a brush length of ∼10 nm in the collapsed state—sufficient to affect solvation without altering transport (Figure 3b).

**Figure 3:**
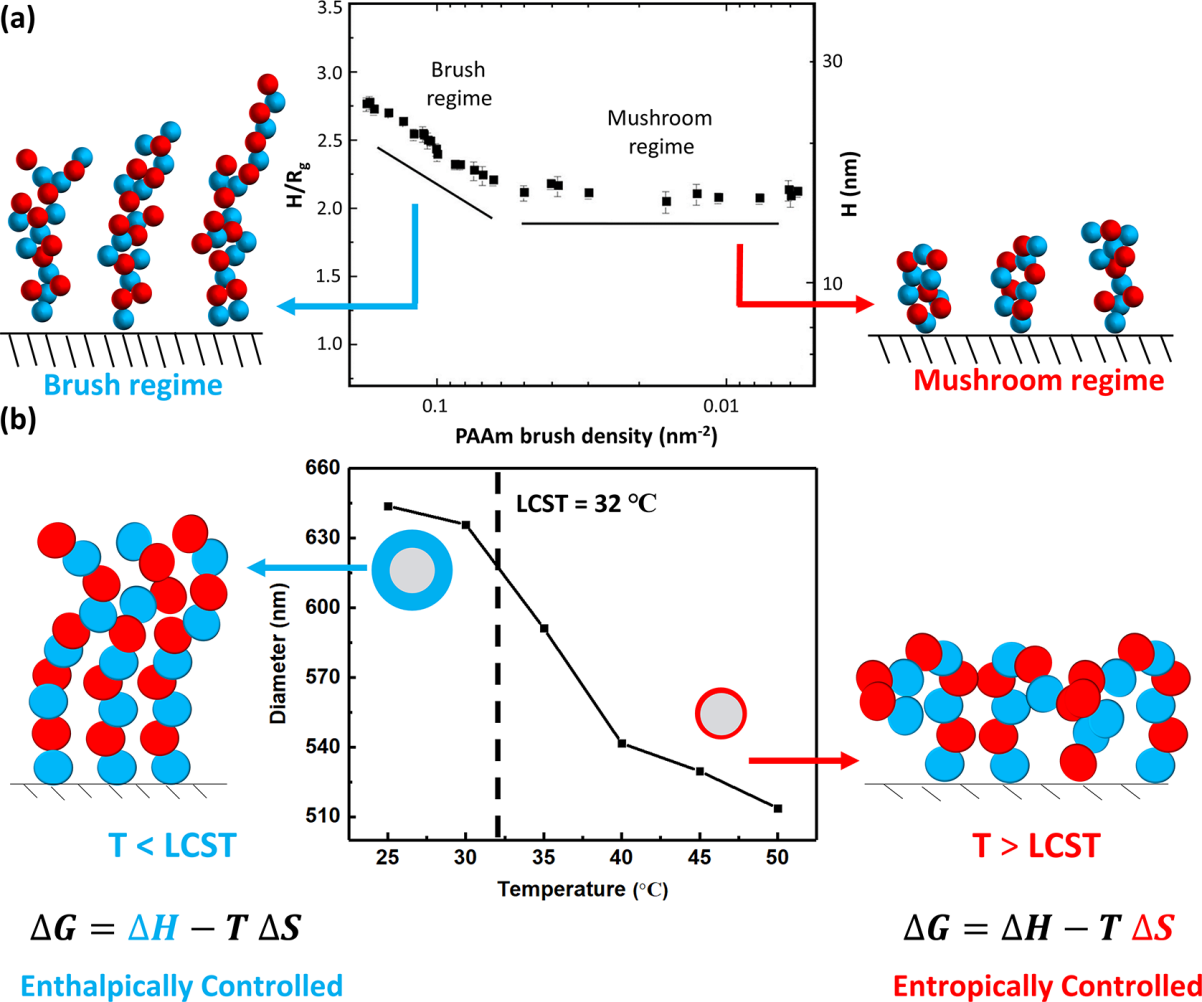
(a) Schematic of brush wet thickness (*H*) and grafting density (σ) on a polymeric brush polymerized on a surface, where *D* represents the distance between grafting sites, ν is dependent on solvent quality (in good solvent, ν ≈ 1/3; in a poor solvent, ν ≈ 0.5), and *R*_g_ is the radius of gyration. Adapted from ref 26. Copyright 2002 American Chemical Society. (b) The size of the silica particles containing p-NIPAM covalently bonded to its surface, as measured by dynamic light scattering (DLS), showcases a large volumetric transition from the solvated to collapsed state as the temperature increases above 32 °C, which corresponds to the LCST of the polymer. Adapted from ref 2. CC BY-NC-ND 4.0.

## Application in the Reduction of Nitrite

We used nitrite (NO_2_^–^) reduction on palladium in water as a model to probe the polymer effects on reaction kinetics. This fast hydrogenation requires only one active site, enabling mechanistic insights. The pathway involves NO* formation from NO_2_^–^ and H_2_, followed by hydrogenation to HNO*, HNOH*, and HN*, leading to NH_3_ or N_2_ depending on surface coverages. In addition, hydroxylamine (NH_2_OH) can also form and readsorb.^28^ Combined with theory, we showed that in water, the reaction proceeds via proton–electron transfer, where protons shuttle through the solvent while electrons flow through the metal, flattening the energy landscape and equalizing barriers for NO* and HNO* reduction. This is crucial, since polymers can modulate proton and water dynamics near the active site.

In the presence of the polymer at low temperature, the reaction order in nitrite (Figure 4d–f) seemed to increase, and at low partial pressures of hydrogen (0.03 bar) it reaches mildly negative orders, while in the Pd/SiO_2_ catalyst (Figure 4a–c) the orders were rather negative.^29^ This would suggest that the coverage decreases at the same concentrations of nitrite in the presence of polymers. In the case of hydrogen, the order decreased even when the polymer was collapsed. This would suggest that the surface coverages are larger. More importantly, the fact that fractional, zeroth, and negative reaction orders are observed at low and high temperatures suggests that the Pd/SiO_2_-p-NIPAM catalyst is not mass-transport-limited, as diffusion is a first-order process. Clearly, the polymer alters the kinetics without inducing a mass transport limitation, which complies with the design rules for stimulus-responsive modulation.

**Figure 4:**
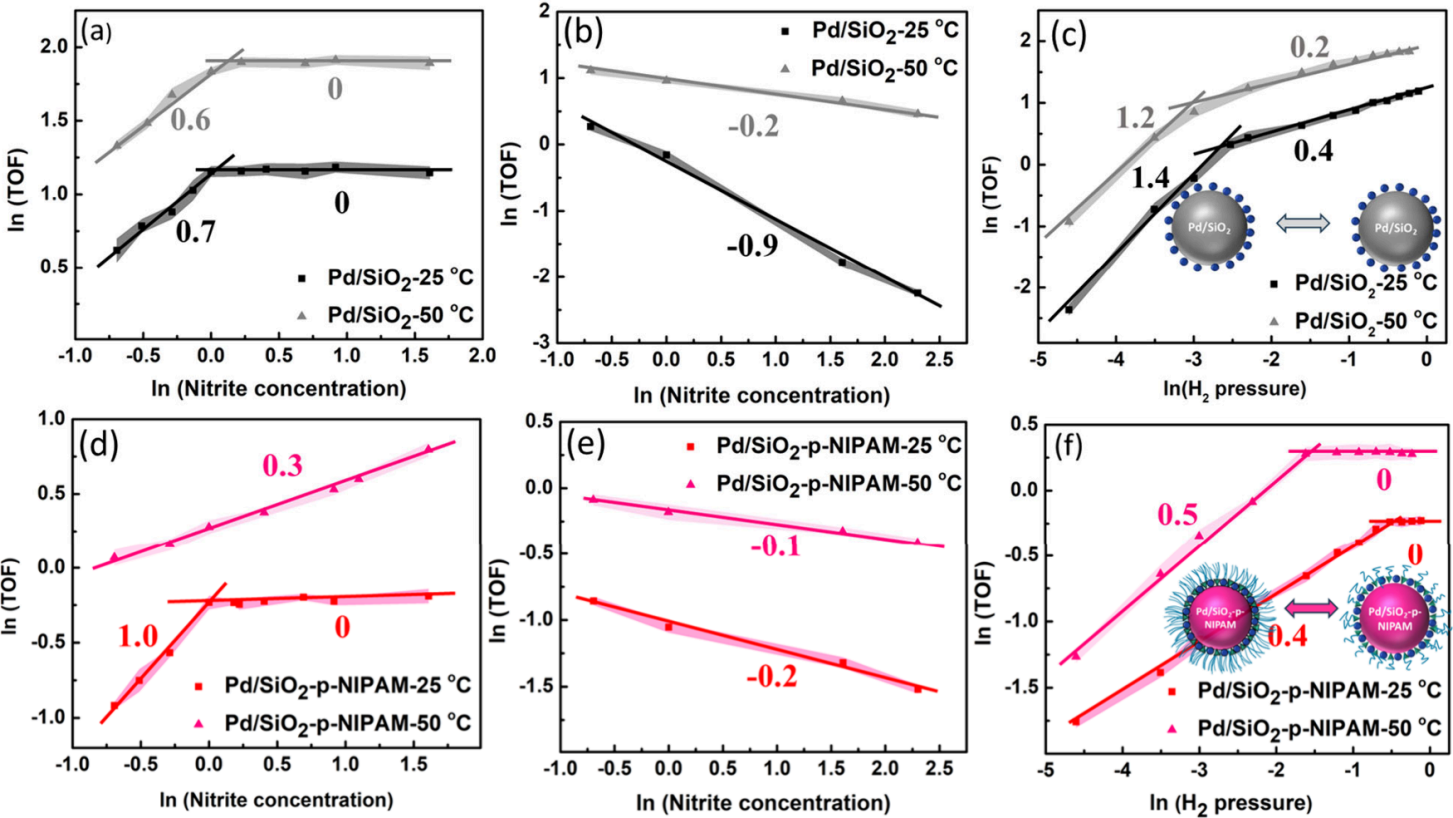
(a, b, d, e) Interplay of nitrite concentration and the TOF using (a, b) Pd/SiO_2_ and (d, e) Pd/SiO_2_-p-NIPAM catalysts with (a, d) 0.8 bar H_2_ and (b, e) 0.03 bar H_2_. (c, f) Impact of hydrogen pressure on the TOF at 1 mM nitrite concentration. Adapted from ref 2. CC BY-NC-ND 4.0.

To understand how the polymer changes the reaction energetics requires the analysis of the rates as a function of the temperature with fixed surface coverages of the key reaction intermediates to ensure that the rate-limiting step and the observed kinetics are not biased by coverage effects. On the uncoated or parent Pd/SiO_2_ catalyst, the apparent barrier was about 36 kJ/mol, which is in line with previous research (Figure 5a). In sharp contrast, the polymer-coated counterpart showed two distinct apparent barriers (Figure 5b). In the low-temperature regime, when the polymer is swollen, the activation energy was ∼11 kJ/mol, while at temperatures above the LCST, the barrier reached values that were closer to those observed on the Pd/SiO_2_ catalyst (∼32 kJ/mol). This is a large decrease in the apparent reaction barrier, which is a dramatic change for a catalytic reaction that already occurs at room temperature.

**Figure 5:**
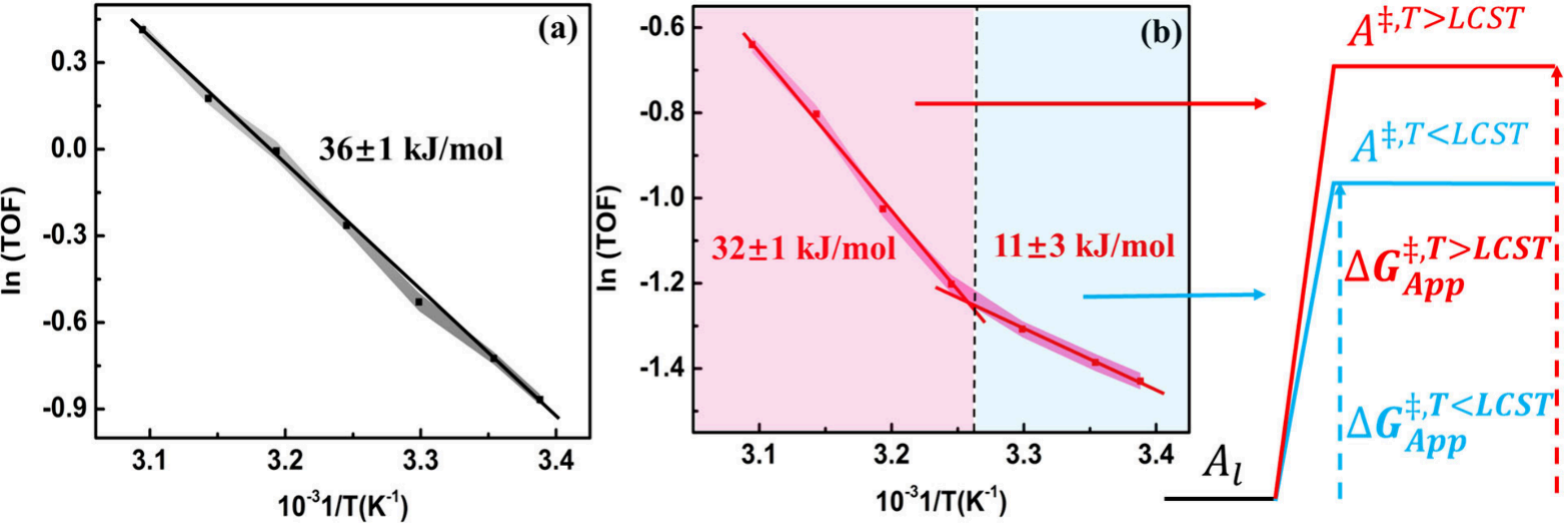
Apparent barriers of activation for the nitrite hydrogenation obtained from the Arrhenius plots of (a) Pd/SiO_2_ and (b) Pd/SiO_2_-p-NIPAM at a 0.03 bar hydrogen pressure and 1 mM nitrite concentration. Adapted from ref 2. CC BY-NC-ND 4.0.

To further investigate the root of this change, one could use transition-state treatments of the reaction rates at the two distinct solvation states of the polymer. Since the full kinetic description of this reaction was known for Pd/SiO_2_, it is possible to explore the impact of the polymer at conditions in which the surface coverage is the same for the low and high temperatures. In this case, the complex rate expression that includes colimiting NO* and HNO* reaction steps can be reduced to eq 15:

15 $$\frac{r}{\left[L\right]}=\frac{k_{B}T}{h}\;\exp\left(\frac{\Delta S_{\mathrm{app}}^{{}^{\circ},⧧}+\Delta S_{\mathrm{app}}^{\varepsilon ,⧧}}{R}\right)\;\exp\left(-\frac{\Delta H_{\mathrm{app}}^{{}^{\circ},⧧}+\Delta H_{\mathrm{app}}^{\varepsilon ,⧧}}{RT}\right){\left[H_{2}\right]}^{3/2}{\left[{{\mathrm{NO}}_{2}}^{-}\right]}^{-1}$$

Here one can use Pd/SiO_2_ to determine the entropy (Δ*S*_app_^°,⧧^) and enthalpy (Δ*H*_app_^°,⧧^) of activation for the transition state at reference conditions, as described in the indirect transduction of stimulus response of polymers to catalytic solvation effects. Once this information is known, it is possible to estimate the excess change in the apparent entropy and enthalpy of activation at temperatures below and above the LCST to interrogate the role of the polymer in the reaction kinetics (Figure 6). The results indicate that at temperatures below the LCST, there is an enthalpic stabilization of *ca.* −25 kJ/mol, reducing the apparent enthalpy of activation to 11 kJ/mol. This stabilization came at the expense of reducing the degrees of freedom of the transition state, as indicated by the excess entropy of activation of *ca.* −89 J mol^–1^ K^–1^, resulting in an apparent entropy of −124 J mol^–1^ K^–1^. At high temperature, the polymer collapsed, and the stabilization was substantially diminished, resulting in apparent enthalpies and entropies of activation that resembled those of the uncoated catalyst.

**Figure 6:**
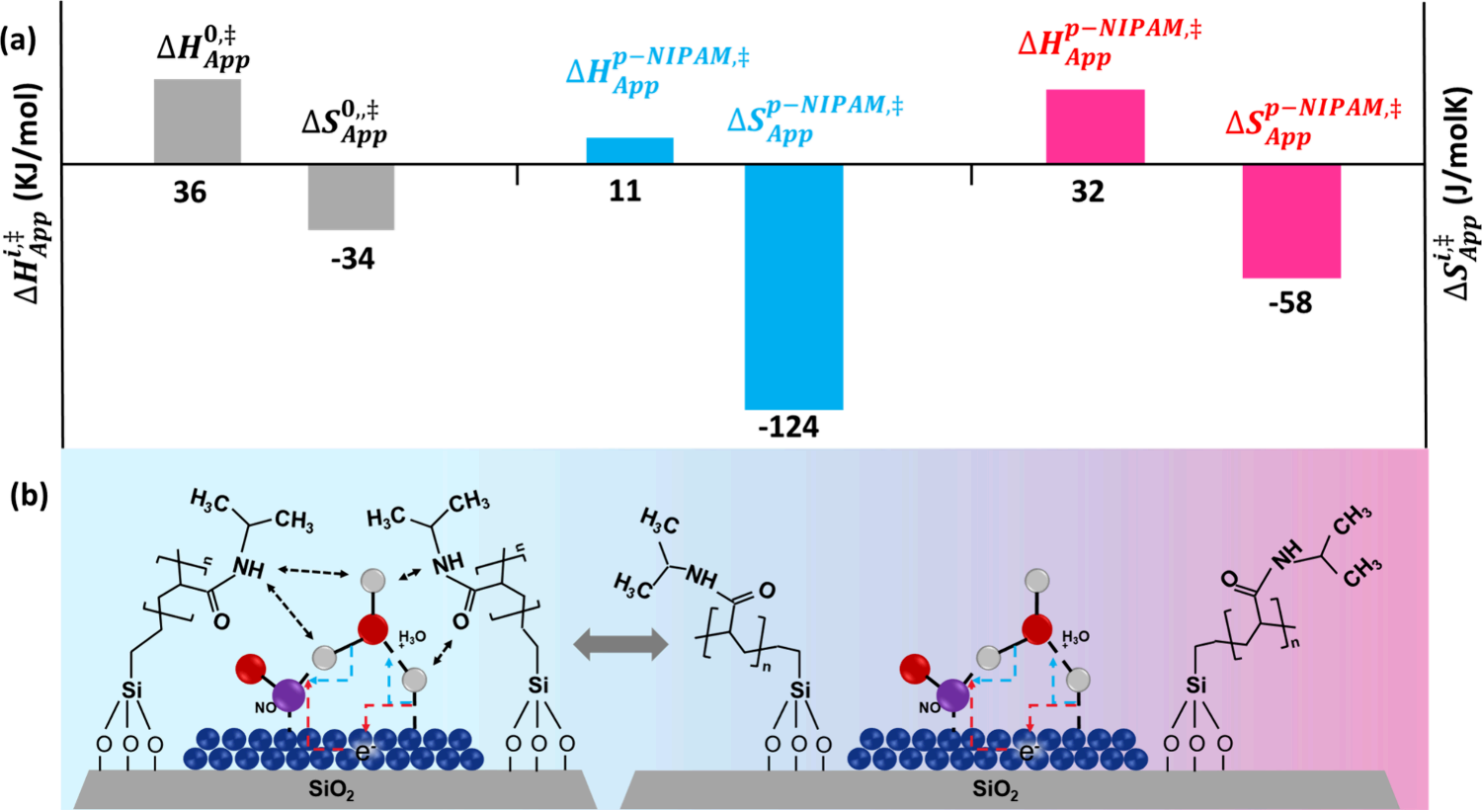
(a) Enthalpy and entropy changes of activation for the Pd/SiO_2_ and Pd/SiO_2_-p-NIPAM catalysts estimated at low and high temperatures. (b) Schematic indicating the change in the polymer–water interactions beyond the LCST. Adapted from ref 2. CC BY-NC-ND 4.0.

The temperature-dependent changes in apparent activation enthalpy and entropy suggest that the microenvironment created by the polymer coating is key to the reaction.^2^ We probed this environment using spectroscopic techniques. First, ^1^H–^1^H nuclear Overhauser effect spectroscopy (NOESY) revealed the polymer’s structural changes. Above the lower critical solution temperature (LCST), the p-NIPAM coating is substantially disordered. Below the LCST, however, the intensity of signals related to N–H interactions between polymer brushes increased 2.5-fold, indicating a more ordered state. Next, diffusion ordered spectroscopy (DOSY) was used to measure the mobility of water within the polymer coating. To ensure that the information is related to the polymer–water interactions, one can use a small amount of water that is equivalent to three layers of water molecules covering the catalyst particles. Above the LCST, water molecules exhibited a single population with a self-diffusion coefficient similar to that of bulk water. In contrast, below the LCST, water mobility decreased approximately 20-fold, revealing two distinct populations: fast-moving and slow-moving water. This bimodal population of water molecules indicates strong hydrogen-bonding interactions between water and the ordered polymer network at low temperatures. Finally, a decrease in the kinetic isotope effect (KIE) with increasing temperature further confirmed that the ordered, water-rich environment at low temperatures plays a crucial role in the catalytic process.

## Application in the Reduction of Nitrobenzene

Next, we explored the usage of these catalysts on nonionic polar molecules to showcase the universal nature of these stimulus-responsive solvation effects. For this purpose, we used nitrobenzene (NB) as a proxy for N–O bond dissociation. Detailed reaction kinetics, catalyst characterization, and DOSY/NOESY NMR experiments indicated that nitrobenzene reduction is colimited by both the formation and the hydrodeoxygenation of phenylhydroxylamine (PHA) to give the aniline (AN) precursor. Transition-state treatment of the kinetics revealed that when the temperature is below the LCST of p-NIPAM (32 °C), the apparent enthalpy of activation decreases 3-fold, following the same trend as observed for the nitrite reduction. At temperatures above the LCST, it was possible to reverse these effects, leading to a similar apparent activation energy as observed in the Pd/SiO_2_ catalyst (Figure 7). These findings unambiguously indicate that these effects can be extrapolated to the reduction of nonionic species.

**Figure 7:**
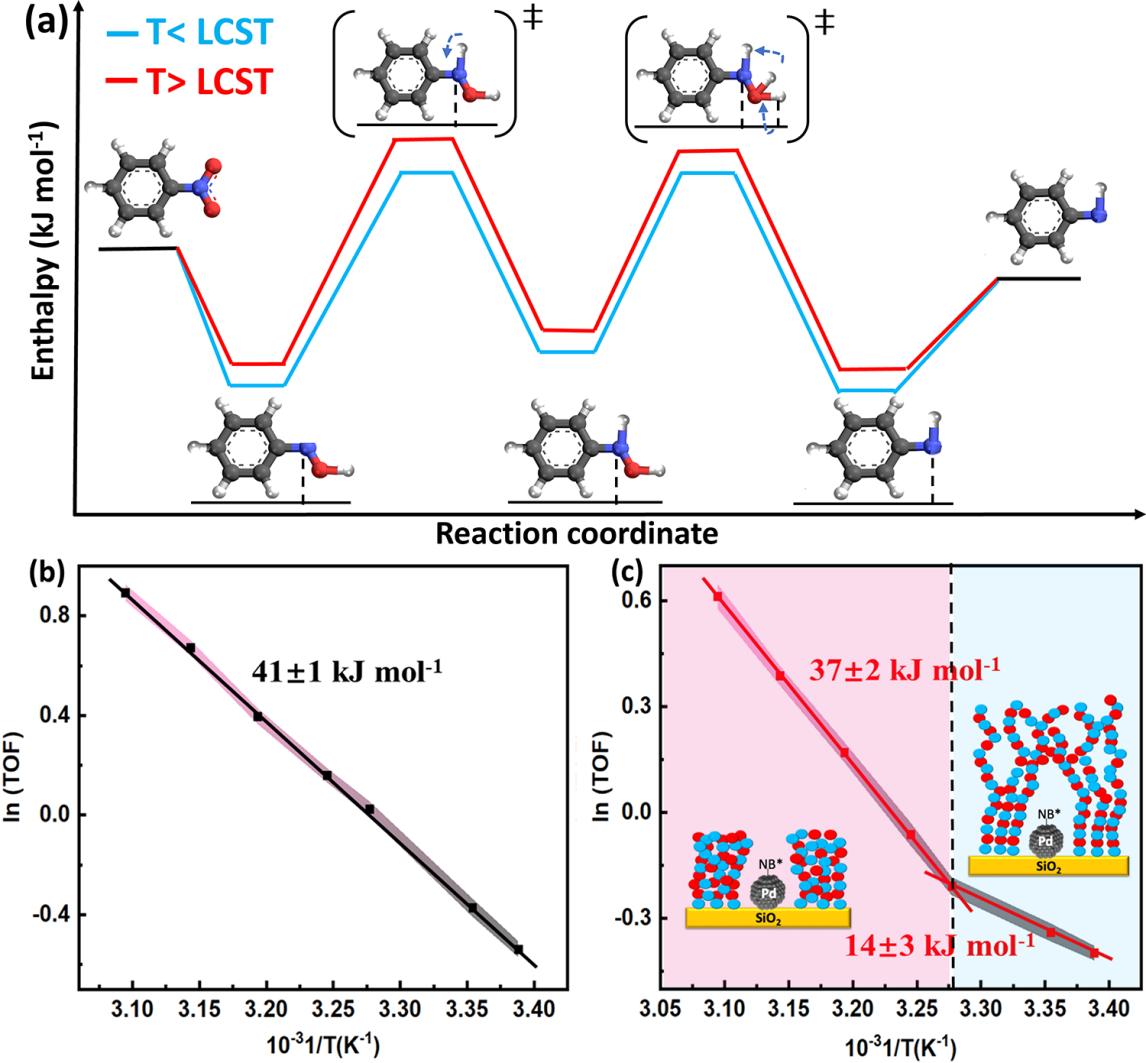
(a) Reaction energy diagram for nitrobenzene hydrogenation to aniline on the p-NIPAM-modified Pd/SiO_2_ catalyst at low and high temperature, indicating the change in the degree of rate control as a function of the reaction conditions. (b, c) Arrhenius plots for the nitrobenzene reduction on (b) Pd/SiO_2_ and (c) Pd/SiO_2_-p-NIPAM catalysts. Adapted from ref 1. CC BY 4.0.

The lower activation barriers observed on p-NIPAM-coated catalysts below the LCST may arise from strong polymer–water interactions affecting proton–electron transfer or from direct polymer–intermediate hydrogen bonding. To distinguish these effects, we performed kinetics in a solvent that stabilizes the polymer and reactants but lacks proton-shuttling activity. Dynamic light scattering showed that in *N*-methyl-2-pyrrolidone (NMP), the volumetric phase transition of p-NIPAM diminished with increasing NMP fraction and disappeared in pure NMP, where the polymer remained solvated at all temperatures (Figure 8a,b). Kinetic studies revealed that in 30 vol % H_2_O/NMP, the apparent barrier was ∼75 kJ/mol for uncoated Pd/SiO_2_ and ∼60 kJ/mol for the coated catalyst, with no changes across the LCST, indicating loss of stimulus-responsiveness (Figure 8d,e). Kinetic isotope effect measurements further showed no rate change with H/D substitution in 30% H_2_O/NMP (blue bars) but a strong rate decrease when both water and hydrogen were replaced by their deuterated counterparts (red bars) (Figure 8c). The fact that the KIE was observed only with D_2_O and D_2_, together with the low activity in aprotic media and the absence of stimulus responsiveness, indicates that water is essential to trigger the solvation effects.

**Figure 8:**
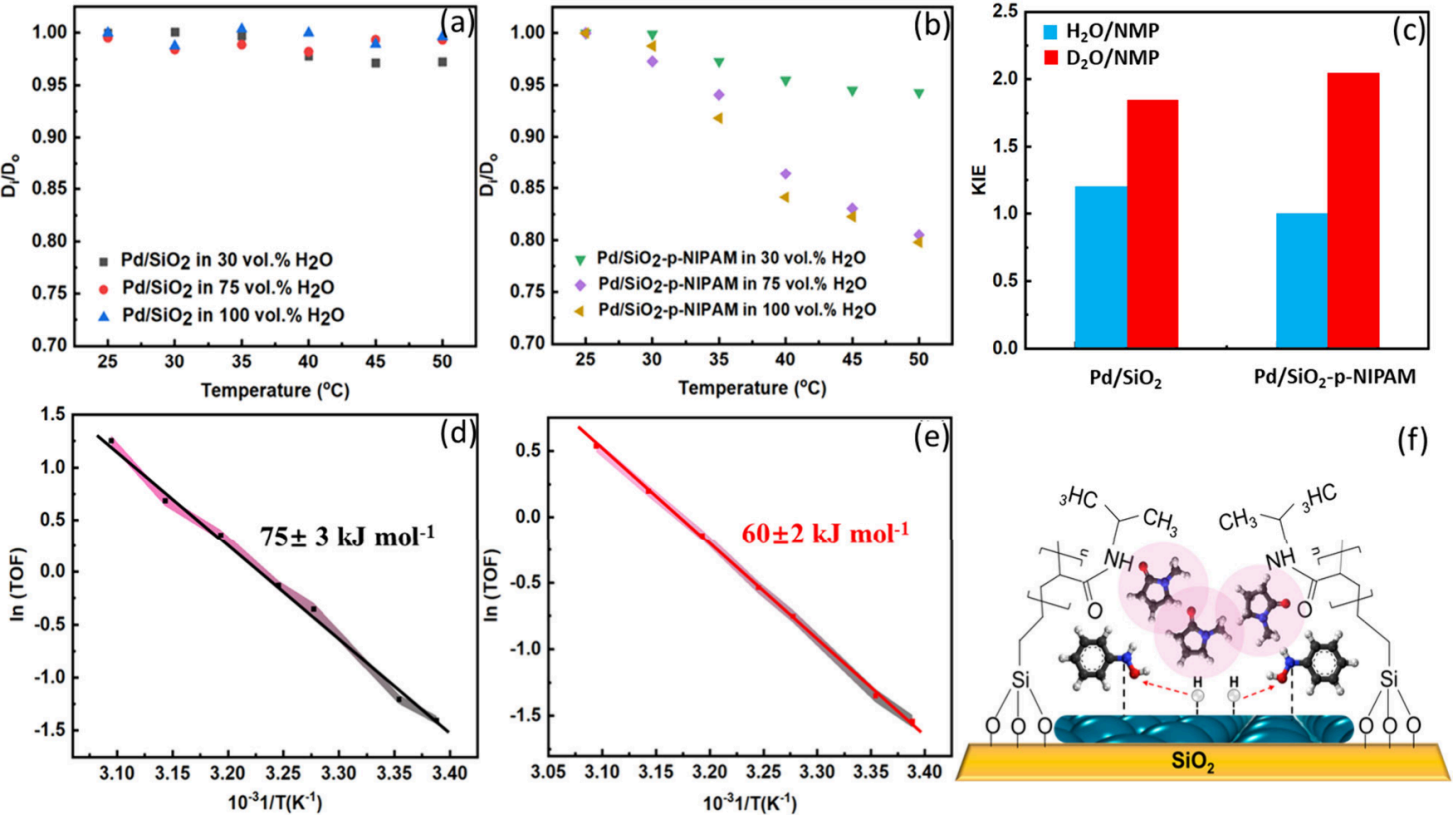
Dynamic light scattering of the (a) Pd/SiO_2_ and (b) Pd/SiO_2_-p-NIPAM catalysts as a function of temperature using concentrations of water that increased from 30 to 100%. (c) Kinetic isotope effect (KIE) study using Pd/SiO_2_ and Pd/SiO_2_-p-NIPAM catalysts. (d, e) Resulting Arrhenius plots for the (d) Pd/SiO_2_ and (e) functionalized Pd/SiO_2_-p-NIPAM catalysts. (f) Schematic illustration of how the direct interaction of NMP with the reactants on the Pd is not sufficient to activate the hydrogenation, as water is needed to conduct the PET reaction. Adapted from ref 3. CC BY 4.0.

These observations indicated that (1) the solvent plays a key role in transferring the hydrogen during the proton–electron transfer reaction, and thus when nonprotonic solvents are employed, the reaction drastically slows down; (2) the utilization of aprotic solvents inhibits the stimulus-responsiveness of the polymer, yielding a solvated coating at temperatures between 20 to 50 °C; and (3) the p-NIPAM does not directly induce the changes in the energy landscape of the reaction, but instead, it is the interaction of the water molecules with the polymer that introduces the enthalpic stabilization of the transition state with the concomitant reduction in the entropy of activation (Figure 8f). This long-range polarization of the water molecules due to the presence of the p-NIPAM is a unique mechanism of actuation that is specific to these stimulus-responsive catalysts.

## Development of Computational Modeling Approaches of Polymer–Catalyst Surface Systems

The complex polymer–catalyst interfaces and their role in determining the reaction kinetics pose a key challenge for multiscale computational modeling. The polymer–solid interface has been investigated using classical force field simulations,^30,31^ which provide rich information on the structural and conformational properties of polymer–solid interfaces. However, to calculate the activation barriers of the elementary steps, first-principles simulations, such as density functional theory calculations combined with transition-state sampling approaches (e.g., nudged elastic band and dimer methods), are required. These calculations provide total energies, which can be corrected to finite temperatures by evaluating the entropic contribution from statistical thermodynamics over simplified models. In addition, QM/MM simulations and newly developed machine-learning force field simulations combined with enhanced sampling approaches can be applied to calculate the free energy profiles; however, these approaches must be benchmarked for each system and are normally associated with a high computational cost. This is particularly true when the solvent is introduced, which complicates the structure and dynamics of the interface.

In our studies, we decided to model the system step by step, starting with the reaction profile over a clean Pd surface with NO_2_^–^ adsorption from the liquid phase, followed by sequential hydrogenation steps to form NH_3_. The free energy calculations, combining DFT-calculated total energies with entropic contributions, were performed to evaluate the stability of surface species and activation barriers for their transformation (Figure 9). The coupling between the reaction intermediates to form precursors to N_2_ was also investigated, but it appears that the activation barrier for the C–C coupling is higher than that for the hydrogenation. Therefore, the experimentally observed high selectivity for N_2_ over Pd likely results from a high coverage of N-containing species on the surface that favors the coupling step. The overall reaction profile shown in the figure suggests that the overall rate is limited by the first couple of hydrogenation steps before forming HNOH because of the comparable energies of transition states from NO to HNO and from HNO to HNOH, which is further supported by experimentally measured reaction order changes in H_2_ as previously explained.

**Figure 9:**
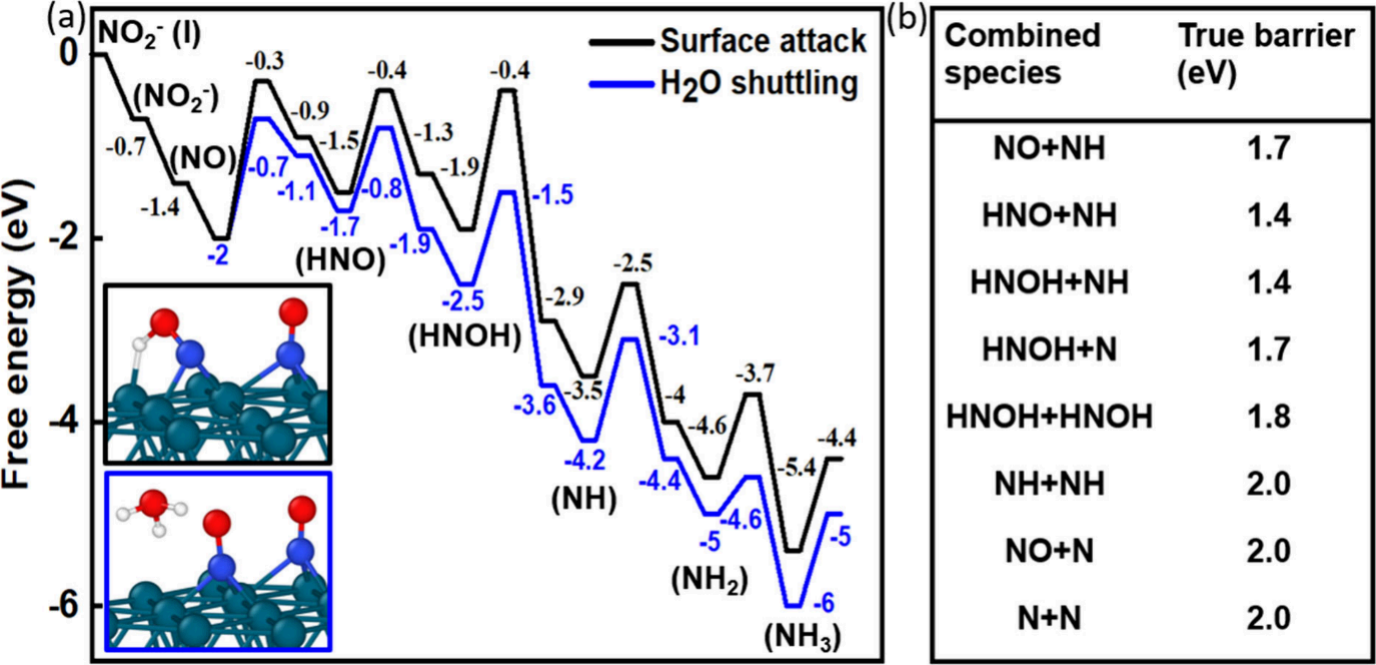
(a) Free energy diagram of NO_2_^–^ reduction on a Pd (111) surface. Black line: hydrogen directly attacks the surface species. Blue line: proton shuttle through H_2_O to the surface species. (b) True barriers for possible N–N coupling species. Reproduced from ref 29. CC BY 4.0.

As the rate-determining step(s) involve water-assisted proton–electron transfer (PET), we hypothesize that the SiO_2_–Pd-polymer interface may regulate the local microenvironment of interfacial water that drives the reaction kinetics to a different extent.^11,12^ In the experiments we conducted, the polymer was instead grown on the silica support without directly binding to the metal catalysts, and therefore, we anticipate that the role of polymer is to directly or indirectly (via water) affect the stability of surface species and kinetics of the hydrogenation. However, p-NIPAM polymers grown on the silica substrate are about 70 nm when swelled below the LCST of 32 °C. This polymer length is well beyond what typical first-principles molecular dynamics simulations and density functional theory calculations can model.

As the polymer is not directly grown on the Pd nanoparticles (metal clusters were about 2 nm), we did not consider the explicit Pd/SiO_2_-p-NIPAM interface. Instead, in a very simplified model, we introduced two NIPAM monomers into the water structure that we adopted from previous work, in which we identified that the rate-determining step for nitrite reduction is the hydrogenation steps after NO formation.^29^ We examined first the hydrogen adsorption energy at the Pd–water interface with or without these two NIPAM monomers by calculating the out-of-plane vibration of hydrogen in extended AIMD simulations (Figure 10). This vibration frequency is centered *ca.* 30 THz, which agrees with the previously measured perpendicular vibrational mode of hydrogen bonded in a threefold hollow site over clean Pd surfaces. Since this frequency did not change much upon the introduction of the NIPAM monomers, one can conclude that the hydrogen binding energy likely remains unchanged. This would be in line with the observation that aprotic solvents cannot induce stimulus-responsive solvation effects, as the proton shuttling is key for these reactions.

**Figure 10:**
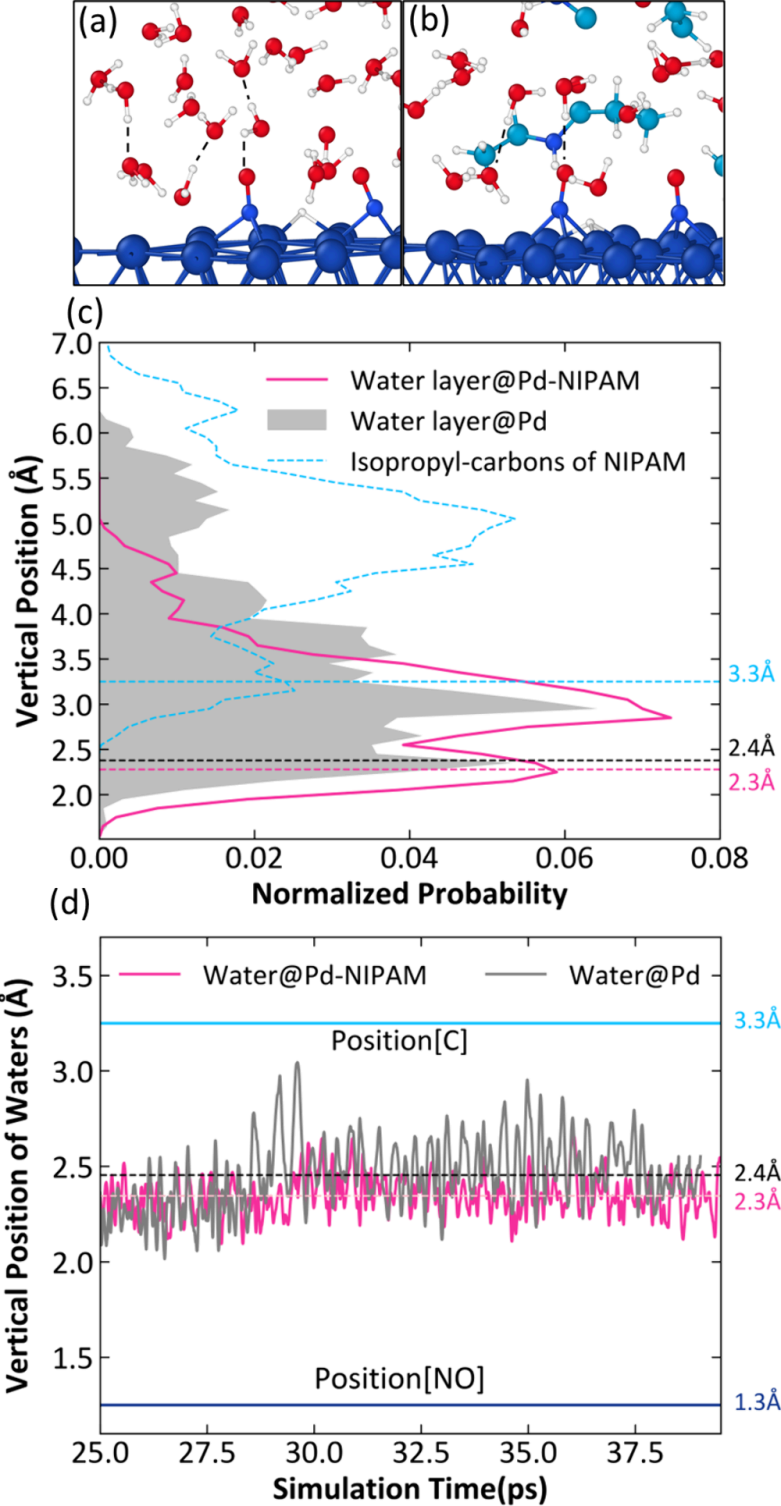
(a, b) Atomic structures of the water interfaces on Pd and Pd-p-NIPAM with surface-adsorbed NO, a key reaction intermediate. Element colors: Pd, deep blue; C, cyan; H, white; O, red; N, blue. (c) Interfacial water layer distribution above the metal surface at the Pd–water interface (shaded area) and Pd-NIPAM–water interface (solid lines). The integral area under each line (area) is equal to 1. The first peak indicates the interfacial water layer. (d) Dynamic interactions of interfacial H_2_O molecules. The dashed line presents the position of the water layer compared to time. The solid line presents the location of one NIPAM monomer, presented by the carbon, and the reactant intermediate NO presented by the O, averaged over AIMD simulations. Reproduced from ref 2. CC BY-NC-ND 4.0.

To explain the experimentally observed lower activation barrier when p-NIPAM swells, we examined the interfacial water structure. Figure 10c presents the water density profile perpendicular to the metal surface. Upon introducing the NIPAM monomer, two main effects emerge: (i) the peak near 5 Å is strongly suppressed, likely due to steric constraints imposed by NIPAM monomers situated at this height above the surface, and (ii) the chemically adsorbed water molecules at the Pd surface shift slightly closer to the metal, also attributable to this confinement. This shortened distance is expected to facilitate proton shuttling through interfacial water, thereby decreasing the activation enthalpy. At the same time, the more compact and structured water layer is perturbed less by proton dynamics, which imposes a higher entropy penalty for the shuttling process, compensating for the reduced activation enthalpy. These results underscore the importance of dynamic atomistic studies to decouple the potential changes in the binding energy of the reactive species prior to the transition state from those induced in the transition state itself.

## Conclusions and Outlook

Creating a living catalyst that can alter the solvation environment and adjust the energy landscape in response to changes in input and output parameters holds great potential for the conversion of complex feedstocks, such as biomass, plastic waste, and wastewater streams, where large variations in feed composition often induce rapid deactivation and byproduct formation when using conventional catalysts. We have shown that p-NIPAM-functionalized catalysts can drastically modify reaction kinetics depending on their state of solvation by altering the molecular arrangement of the solvent rather than on mass transport limitations. This unique operating mechanism enables finer tuning of chemical kinetics and product selectivity in comparison to previously reported methodologies.

The key lies in tailoring the mechanochemical response of the polymer coating to the desired catalytic performance at both the solvated and collapsed states of the polymer. We are convinced that coupling detailed reaction kinetics with dynamic molecular-based modeling will improve our understanding of model systems, which can serve as cornerstones for synthesizing catalysts with self-regulating and self-maintaining capabilities capable of operating in complex, dynamic environments. Achieving this requires integrating advanced polymer chemistry with physical chemistry to unravel the chemo-physical response of the polymer prior to its incorporation into the catalyst. Such a chemo-physical response not only perturbs the energy of the interfacial system but also leads to a considerable entropic contribution; this compensation in free energy change can be described through enhanced sampling approaches combined with QM/MM simulations or machine-learning force field simulations. Also, this change in free energy of the system likely happens at distinct time scales of the polymer phase transition, evolution of solvent configuration, and reaction elementary steps, and these dynamics also correlate with each other and are challenging to be decoupled. Therefore, multiscale simulations are valuable to reveal the interplay between these different degrees of freedom from a relatively slower, macroscopic polymer phase transition to much faster chemical reactions at the atomic scale. This knowledge can be leveraged to create multifunctional cooperative catalysts and catalytic cascading systems, similar to the cellular biomachinery of living organisms, which can conduct multiple reaction steps in a single reactor. For instance, pH-responsive polymers can enable selective conversion in reactions, such as glucose isomerization to fructose followed by acid-catalyzed dehydration to 5-hydroxymethylfurfural. By exploiting stimulus-responsive behavior, one could envision catalysts that, upon reaching full conversion, enter a new operational window to initiate a second conversion step, continuing a programmed routine until the final product is generated. Materials capable of switching ON and OFF could enable the development of truly smart chemical plants that convert wastewater into value-added chemicals, biomass into key fuels and intermediates, and harmful emissions into benign products. Important considerations in designing these materials are their stability in the reaction medium and the catalyst selectivity, as rapid deactivation or low yields could render the process unfeasible.

Finally, the vast library of organic chemistries available for these materials can facilitate the synthesis of multistimuli-responsive catalysts that can be triggered by pressure, pH, temperature, light, or magnetic fields, thereby providing multiple noninvasive activation strategies for remote operation. To realize this vision, it will be essential to combine polymer chemistry, comprehensive kinetic testing, and molecular modeling.

## Abbreviations

LCST: lower critical solubility temperature
p-NIPAM: poly­(*N*-isopropylacrylamide);
